# The Effects of an Infant Formula Enriched with Milk Fat Globule Membrane, Long-Chain Polyunsaturated Fatty Acids and Synbiotics on Child Behavior up to 2.5 Years Old: The COGNIS Study

**DOI:** 10.3390/nu12123825

**Published:** 2020-12-15

**Authors:** Ana Nieto-Ruiz, Estefanía Diéguez, Natalia Sepúlveda-Valbuena, Florian Herrmann, Tomás Cerdó, Francisca López-Torrecillas, Roser De-Castellar, Jesús Jiménez, Miguel Pérez-García, María T. Miranda, Andrés Catena, José A. García-Santos, Mercedes G. Bermúdez, Cristina Campoy

**Affiliations:** 1 Department of Paediatrics, School of Medicine, University of Granada, Avda. Investigación 11, 18016 Granada, Spain; ananietoruiz@gmail.com (A.N.-R.); estefaniadieguezcastillo@gmail.com (E.D.); sepulveda.natalia@hotmail.com (N.S.-V.); dr.f.herrmann@gmail.com (F.H.); thomascr3@hotmail.com (T.C.); joseantonio_gsantos@outlook.es (J.A.G.-S.); mgbermudez@ugr.es (M.G.B.); 2 EURISTIKOS Excellence Centre for Paediatric Research, Biomedical Research Centre, University of Granada, 18016 Granada, Spain; 3 Instituto de Investigación Biosanitaria ibs. GRANADA, Health Sciences Technological Park, 18012 Granada, Spain; 4 Mind, Brain and Behaviour Research Centre-CIMCYC, University of Granada, 18011 Granada, Spain; fcalopez@ugr.es (F.L.-T.); mperezg@ugr.es (M.P.-G.); acatena@ugr.es (A.C.); 5 Nutrition and Biochemistry Department, Faculty of Sciences, Pontificia Universidad Javeriana, 110231 Bogotá, Colombia; 6 Department of Personality, Evaluation and Psychological Treatment, School of Psychology, University of Granada, 18011 Granada, Spain; 7 Ordesa Laboratories, S.L., 08820 Barcelona, Spain; Roser.DeCastellar@ordesa.es (R.D.-C.); Jesus.Jimenez@ordesa.es (J.J.); 8 Department of Biostatistics, School of Medicine, University of Granada, 18016 Granada, Spain; tmiranda@ugr.es; 9 Spanish Network of Biomedical Research in Epidemiology and Public Health (CIBERESP), Granada’s Node, Institute of Health Carlos III, 28029 Madrid, Spain

**Keywords:** infant formula, milk fat globule membrane, long-chain polyunsaturated fatty acids, bioactive compounds, children behavior, breastfeeding

## Abstract

Although early life nutrition influences brain development and mental health, the long-term effects of supplemented infant formula on children´s behavior remain unclear. We analyzed the effects of a bioactive nutrients-enriched-infant formula on children’s behavior up to 2.5 years, compared to a standard infant formula or breastfeeding. Current analysis involved 70 children who were fed a standard infant formula (SF, *n* = 29) or a bioactive compounds enriched-infant formula (EF, *n* = 41), during their first 18 months of life, and 33 breastfed (BF) children (reference group) participating in the COGNIS study. Behavioral problems were evaluated using the Child Behavior Checklist at 18 months and 2.5 years. Different statistical analyses were performed using SPSS. EF children aged 2.5 years presented fewer pathological affective problems than SF children. Besides, SF children were classified more frequently as bordering on internalizing problems than BF children. Rates of externalizing problems were increased in SF infants compared to EF and BF infants. Higher maternal IQ was found to have beneficial effects on internalizing and total problem rate in their offspring at 18 months of life; finally, higher maternal educational level was related with fewer ADHD problems in children at 18 months, as well as internalizing, externalizing, total and anxiety problems in children aged 2.5 years. Our analysis suggests that enriched infant formula fed infants seem to show fewer behavioral problems up to 2.5 years compared to a standard infant formula-fed infants. In addition to type of early feeding, maternal IQ and educational level seem to play a key role on children behavioral development.

## 1. Introduction

Brain development is particularly vulnerable to adverse prenatal and postnatal early-life events [1]. Among them, inadequate nutrition during the first 1000 days of life may have a negative influence on brain growth and functional development, including cell proliferation, synaptogenesis and myelination [2], thereby affecting cognitive development and behavioral performance later in life [3,4].

Breastfeeding is the gold standard for infant feeding due to its multiple short- and long-term health benefits for both child and mother [5]. In fact, breastfeeding confers protection against breast and ovarian cancer and contributes to birth spacing; it might also protect against type 2 diabetes in lactating mothers. Moreover, human milk provides protection against child infections and probably reduces the risk of developing overweight and diabetes along life [6]. There is also scientific evidence for the positive effects of breastfeeding on cognitive function [7], although its beneficial effects on emotional regulation and behavior development still remain unclear [8,9,10,11]. Conversely, the use of infant formula for non-breastfed or partially breastfed infants is widely extended in high income countries, probably related to socioeconomic circumstances, such as educational level or employment status [6]. In light of current advances in food technologies, infant formulas are being continuously improved trying to resemble human milk in terms of optimal children’s growth and neurodevelopment [12]. Long-chain polyunsaturated fatty acid (LC-PUFA) enriched formulas have been approved and recommended by international authorities [13] because of their association with better visual acuity and cognitive development later in life [14,15,16]. However, potential long-term effects of these type of infant formulas on neurodevelopment and behavior are a current matter of discussion [17].

There is also an increasing scientific interest in infant formula enriched with milk fat globule membrane (MFGM) [18,19,20], a complex membrane structure composed of proteins (1–4% of the total content of milk protein), enzymes and lipids [21]. Timby et al. found that infants fed with an MFGM-supplemented infant formula showed higher scores in cognitive domains than those fed with a standard formula at 12 months of age [22]. Furthermore, a study performed in preschool children aged 2.5–6 years, who daily received phospholipid-rich MFGM formula over four months, showed reduced scores in internal, external and total behavioral problems [23].

Other important bioactive components of human milk are pre- and probiotics, which facilitate a healthy structural and functional development of gut microbiota in the offspring [24,25]. It is known that intestinal microbiota might contribute to neural network shaping and response to neurotransmitters, probably modulating brain development and neurocognitive function [26,27].

Until now, most studies have demonstrated the beneficial effects of one single bioactive compound supplementation in infant formulas [22]. However, new infant formulas are currently developed based on the addition of different human milk-like bioactive compounds, including LC-PUFAs, MFGM, synbiotics (pre- and probiotics), as well as human milk oligosaccharides, sialic acid, nucleotides, or gangliosides, among others. It is complicated to evaluate the effect of a single nutrient, so possible beneficial effects might be due to the synergistic effect of all formula components. Bearing in mind these considerations, the current study analyzed the effects of an infant formula supplemented with specific functional nutrients on behavioral development in healthy children up to 2.5 years old, compared to those fed with a standard formula or human milk.

## 2. Materials and Methods

### 2.1. Study Design and Subjects

The COGNIS study (A Neurocognitive and Immunological Study of a New Formula for Healthy Infants) is a prospective, randomized double-blind, nutritional intervention study, registered at www.ClinicalTrials.gov, Identifier NCT02094547. The primary outcome of the COGNIS study was the neurocognitive development of children up to 6 years old, while secondary outcomes included behavior and temperament, infant growth, infectious and allergic events, among others. Detailed study design has been published elsewhere [28,29]. Briefly, 220 healthy full term babies were involved in the study; from these, 170 infants aged between 0–2 months old were randomized using a mathematical statistical method (ratio 1:1) to receive, during their first 18 months of life, either a standard infant formula (SF *n* = 85), or an experimental infant formula (EF *n* = 85) containing MFGM components [10% of total protein content (wt:wt)], synbiotics [Fructooligosaccharides: Inulin, proportion 1:1; *Bifidobacterium longum* subsp. *infantis* CECT7210 (*Bifidobacterium infantis* IM1) and *Lactobacillus rhamnosus* LCS-742], LC-PUFAs, gangliosides, sialic acid and nucleotides. Both infant formulas were provided by Laboratorios Ordesa, S.L. (Barcelona, Spain), and were developed according to the guidelines of the Committee on Nutrition of the European Society for Paediatric Gastroenterology, Hepatology and Nutrition (ESPGHAN) [30], as well as international and national recommendations for the composition of infant formulas (Table S1). Infants received initiation formula up to 6 months of age; follow-on formula was given between 6–18 months of age. Infants who were included in both formula groups received 30 days maximum of exclusive breastfeeding, followed by exclusive or >70% daily formula intake. As a reference group, 50 exclusively breastfed (BF) infants for at least 2 months, were included between 0–6 months of age.

Inclusion criteria: eligible infants were healthy term infants [37–41 weeks gestational age (GA)], with adequate birth weight for GA (between 3–97 percentile), normal APGAR score (7–10) and umbilical pH ≥ 7.10. They must have had the availability to continue throughout the study period, and parents or legal guardians had to have signed the informed consent.

The exclusion criteria were defined as follows: infants participating in another study; infants who suffered neurological disorders (hydrocephalus, perinatal hypoxia, intraventricular hemorrhage, neonatal meningitis, septic shock, West Syndrom,…) or gastrointestinal disturbances (mainly cow’s milk protein allergy/intolerance or lactose intolerance); maternal pathological background (neurological diseases, mental illness, metabolopathies (type 1 diabetes mellitus), chronic diseases (hypothyroidism), maternal malnutrition or prenatal infections (TORCH complex,..)), mothers who received drug treatments during pregnancy or lactation which are potentially harmful for neurodevelopment (anxiolytics, antidepressants,…); or parents’ impossibility to continue through the study.

Regarding withdrawal criteria, those infants who met the following criteria after randomization were excluded from the study: infants fed with another infant formula (different from SF or EF) for a week or more; breastfed infants with formula intake >25% before 6 months; formula fed infants with human milk intakes higher than 25% beyond the third month of life; any adverse event that could interfere with study follow-up, cow’s milk protein allergy/intolerance or lactose intolerance, infant formula intake rejection or neurological disorder.

**Figure 1:**
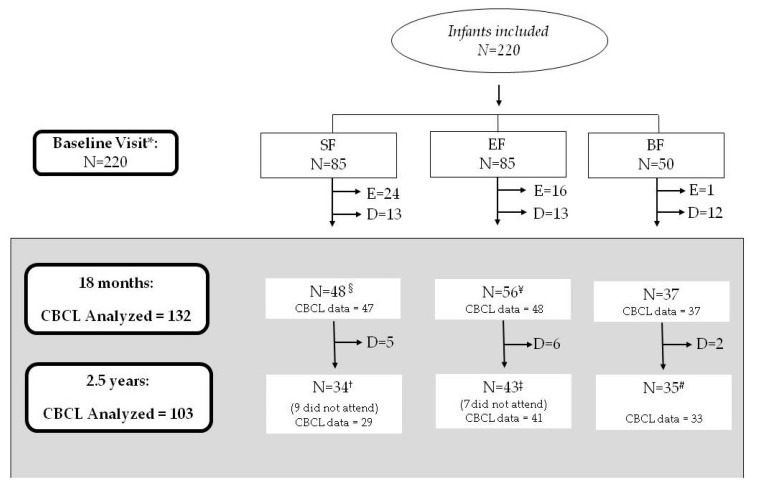
Participant flowchart from recruitment to 2.5 years old. SF, standard infant formula; EF, experimental infant formula; BF, breastfed infants; D, drop-outs; E, exclusions, N, sample size. Up to 18 months of life, a total of 40 infants were excluded in the SF and EF groups as follows: 24 were excluded in the SF group (1 infant due to perinatal hypoxia, 1 infant had growth deficiency, 15 infants met the withdrawal criteria referring to formula intake, 2 infants were colic, 3 were excluded due to lactose intolerance, 1 infant was due to digestive surgical intervention, and 1 infant suffered hydrocephalus); 16 infants were excluded in the EF group (2 infants presented growth deficiency, 2 infants lactose intolerance, 11 infants met the withdrawal criteria referring to formula intake, and 1 was excluded due to epileptic seizure). Furthermore, one infant of the BF group was excluded because they were not breastfed. * BF infants were randomized between 0–6 months of age. ^§^ 1 mother did not fill the child behavior checklist (CBCL) test at 18 months old; ^¥^ 8 mothers did not complete the CBCL test at 18 months old; ^†^ 5 mothers did not fill the CBCL test at 2.5 years old; ^‡^ 2 mothers did not fill the CBCL test at 2.5 years old; ^#^ 2 mothers did not complete the CBCL test at 2.5 years old. Sixteen children at 2.5 years old did not show up at the evaluation (described as “did not attend”). During the follow-up visit at 18 months, dropouts were considered those who did not continue to participate in the study: mainly due to not wanting to continue in the study or not being able to come to the follow up visits, usually for a change in place of residence or workplace conditions of the parents.

The current study involved 132 infants at 18 months of life (SF = 47; EF: 48; BF = 37) and 103 children at 2.5 years old (SF = 29; EF: 41; BF = 33). A detailed flow chart of the recruited participants up to 2.5 years old is shown in Figure 1.

### 2.2. Ethics, Consent, and Permissions

This study was performed according to the updated Declaration of Helsinki Principles [31], and all procedures were approved by the Research Ethical Committee of the University of Granada and the Bioethical Committees for Clinical Research of the University San Cecilio and Mother–Infant Hospitals in Granada (Spain). All families were informed about all procedures during the follow-up and a signed informed consent was obtained from each parent or legal guardian before involving each child in the study.

### 2.3. CBCL Test (Child Behavior Checklist)

To evaluate child behavior and psycho-emotional disorders at 18 months and 2.5 years old, the Child Behavior Checklist (CBCL) (validated Spanish version for 1.5–5 years), was filled out by parents or caregivers [32]. This questionnaire is composed of 101 items divided into two scales (internalizing and externalizing problems) and a total problems scale. The internalizing problems scale contains four syndromic subscales: emotionally reactive, anxious/depressed, somatic complaints and withdrawn. The externalizing problems scale is composed of two syndromic subscales: attention problems and aggressive behavior. In this study, externalizing and internalizing scales were used as substitutes for syndromic scales. Furthermore, the CBCL test also measures the following diagnostic and statistical manual of mental disorders (DSM)-oriented scales: affective problems, anxiety problems, pervasive developmental problems, attention deficit hyperactivity disorders (ADHD) and oppositional defiant problems. A Likert-type scale is used, with responses 0 (“not true”), 1 (“somewhat or sometimes true”) or 2 (“very true or often true”), taking into account the two months previous to the assessment. The total direct score on each scale was obtained automatically with Achenbach system of empirically based assessment (ASEBA) software. In order to perform a longitudinal study, direct scores on the total, externalizing, internalizing, and DSM-oriented scales were used (mean normative values: *total problems* = 33.5; externalizing problems = 14; internalizing problems = 9; affective problems = 2.2; anxiety problems = 3.5; pervasive developmental problems = 3; ADHD = 5.5, and oppositional defiant problems = 4 [33]. Additionally, typical scores (T) in each scale were divided, according to CBCL test standards, into three categories: normal (no behavioral problems, below the 93rd percentile: T < 60), borderline to clinical range (behavioral problems, 93rd to 97th percentiles: 60 ≤ T ≤ 63) and clinical/pathological (behavioral problems, above the 97th percentile: T ≥ 64) [33,34].

### 2.4. Statistical Analysis

All statistical analyses were performed using IBM^®^ SPSS Statistics^®^ program, version 22.0 (SPSS Inc., Chicago, IL, USA). Normally distributed variables were expressed as the mean ± standard deviation (SD), and non-normal variables as the median and interquartile range (IQR). Categorical variables were presented as frequencies and percentages. Differences in CBCL scores among SF, EF and BF groups were contrasted using Chi-squared or Fisher’s exact tests for categorical variables, depending on the number of subjects in the analysis. In addition, analyses of group comparisons using a one-way analysis of covariance (ANCOVA) including maternal educational level and IQ, smoking during pregnancy, paternal educational level, place of residence and socioeconomic status as confounders, were performed. Furthermore, a generalized linear mixed model (GLMM) for repeated measures was developed to identify longitudinal differences between study groups. Bonferroni-corrected post-hoc comparisons were used to identify significant pair-wise group differences (corrected *p* value < 0.05).

Finally, a Wald test for logistic regression was performed to evaluate the effects of potential confounders on the likelihood (odds ratios (ORs) and 95% confidence interval (CI)) of having Borderline/Clinical values in the CBCL test. At 18 months, logistic regression models were performed, including one CBCL outcome as a dependent variable (internalizing, externalizing and total problems; affective, anxiety, pervasive developmental, ADHD and oppositional defiant problems) and the following confounder variable list as independent variables: maternal educational level and IQ, smoking during pregnancy, paternal educational level, place of residence, and the COGNIS groups. At 2.5 years old, logistic regression models were accomplished including the same CBCL outcomes mentioned above as dependent variables, and maternal educational level, socioeconomic status, place of residence and the COGNIS groups as independent variables. *p* values < 0.05 were considered statistically significant.

Statistical power was calculated for the current study applying the following equation [35]:

(1) $$n\;=2\;{\left(\frac{Z_{\alpha }\;+\;Z_{2\beta }}{\delta^{\prime }}\right)}^{2}.$$

## 3. Results

### 3.1. Characteristics of the COGNIS Study Participants up to 2.5 Years Old

Background and baseline characteristics of parents and children are shown in Table 1. At 18 months of life, BF infants’ mothers presented a higher educational level (*p* < 0.001) and IQ (*p* = 0.030) compared to mothers of both formula groups, and EF infants, respectively. Moreover, a higher percentage of the SF group mothers smoked during pregnancy with respect to mothers of the BF group (*p* = 0.049). Paternal educational level was also higher in the BF group compared to the EF group (*p* = 0.013); BF participants more frequently resided in rural areas (*p* = 0.037). At 2.5 years, significant differences in maternal educational level, place of residence and socioeconomic status were found between study groups. In fact, mothers of BF infants showed a higher educational level (*p* = 0.001) with respect to mothers of both infant formula groups. SF participants more frequently resided in urban areas than BF infants (*p* = 0.012). Concerning socioeconomic status, those parents whose children were breastfed had higher status compared to EF-fed infants´ parents (*p* < 0.001). Due to the COGNIS study design, days of breastfeeding significantly differed between BF and formula study groups (*p* < 0.001). No other significant differences in baseline characteristics were found in parent–child pairs participating in the COGNIS study.

In order to reduce the possibility that selection bias could affect study conclusions, we analyzed background variables in the missing sample (i.e., those children who were excluded or withdrew from the study during the follow-up time). It is noteworthy that at the beginning of the study there were no differences between infant formula groups regarding baseline characteristics, as previously reported [25]. Considering drop-out subjects, differences among COGNIS groups were only found in maternal educational level: mothers of BF infants presented higher educational level compared with SF and EF infant’s mothers. Moreover, the exclusion rate was similar in both infant formula groups, being statistically different compared to the BF group (data not shown).

### 3.2. Effects of Type of Early Nutrition on Behavior Development in COGNIS Infants at 18 Months and 2.5 Years Old

Table 2 shows the association between the type of feeding and CBCL scores, categorized in each scale into normal, borderline, and pathological outcomes according to the standard test. No differences were found between COGNIS groups at 18 months of life. However, at 2.5 years old, SF-fed children were classified more frequently as borderline on internalizing problems than BF children (SF: 24.1%; BF: 3.0%; *p* = 0.042). Moreover, EF children less frequently presented clinical pathological affective problems compared to SF-fed children at 2.5 years old (EF: 0.0%; SF: 13.8%; *p* = 0.026). Overall, the percentage of EF children who were classified as normal behavior was similar to that of the BF children.

We next evaluated the effects of type of feeding on CBCL scores in infants at 18 months and 2.5 years old (Table S2). At 18 months of life, no differences were found between SF, EF, and BF infants. Nevertheless, at 2.5 years old, results showed that children who received SF presented higher scores in internalizing (*p* = 0.035) and total problems (*p* = 0.017), as well as ADHD (*p* = 0.039), compared to those who were breastfed. Interestingly, children who received EF or BF showed lower scores in externalizing problems (*p* = 0.005) than SF-fed children. Overall, CBCL scores did not differ between children who received EF and breastfed infants. However, those significant differences did not remain after adjustment for maternal educational level, socioeconomic status and place of residence.

**Figure 2:**
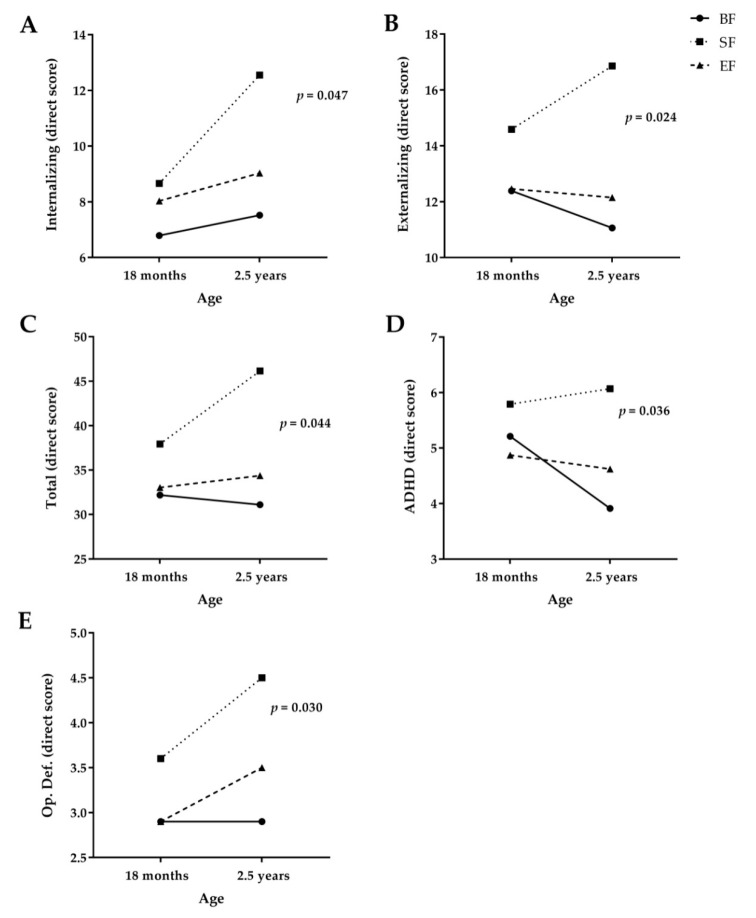
Longitudinal study of CBCL Scales at 18 months and 2.5 years old. ADHD, attention deficit/hyperactivity disorders; BF, breastfeeding; SF, standard infant formula; EF, experimental infant formula; Op. Def, oppositional defiant. Generalized linear mixed model of repeated measures for direct scores for Internalizing Problems (**A**), Externalizing Problems (**B**), Total Problems (**C**), ADHD (**D**), and Oppositional Defiant Problems (**E**) in COGNIS participants. Mean normative values: total problems = 33.5; externalizing problems = 14; internalizing problems = 9; ADHD = 5.5 and oppositional defiant problems = 4 [33].

Afterwards, a longitudinal study of behavior development up to 2.5 years of age was conducted using a GLMM of repeated measures (Table 3 and Figure 2). Significant differences were found between study groups in internalizing, externalizing, total, ADHD, and oppositional defiant problems (Table 3). As shown in Figure 2, SF children presented increased rates in internalizing (*p* = 0.047) (Figure 2A), total (*p* = 0.044) (Figure 2C), ADHD (*p* = 0.036) (Figure 2D), and oppositional defiant problems (*p* = 0.030) (Figure 2E) compared to BF children during their first 2.5 years of age. Interestingly, our results seemed to suggest a similar score increase between EF children and BF children. In addition, children fed with SF showed a significantly higher score increase in externalizing problems than those fed with EF or BF (*p* = 0.024) (Figure 2B). It is important to note that lower CBCL scores are associated with better behavior development, i.e., fewer behavioral problems.

### 3.3. Effects of Potential Confounders on Behavioral Development in COGNIS Infants at 18 Months and 2.5 Years

Finally, a Wald test for logistic regression was performed in order to evaluate the influence of other confounding variables on child behavioral development. At 18 months, the model included maternal educational level and IQ, smoking during pregnancy, paternal educational level, place of residence, and the study group (Table S3). Higher maternal IQ was associated with a decreased risk of internalizing [OR: 0.945 (95% CI: 0.90–60.986); *p* = 0.010] and total problems [OR: 0.965 (95% CI: 0.93–21.000); *p* = 0.048] in their offspring. Moreover, lower maternal educational level (secondary educational level) was related to an increased risk of infants suffering ADHD problems [OR: 6.857 (95% CI: 1.135–41.432); *p* = 0.036]. On the other hand, maternal educational level, socioeconomic status, place of residence and the study groups were included in the Wald test for logistic regression model at 2.5 years old (Table S4). Maternal educational level was positively associated with behavioral development in their offspring at 2.5 years old: children whose mothers had a lower educational level (primary educational level) showed an increased risk of suffering behavioral problems, including internalizing [OR: 7.125 (95% CI: 1.857–27.341); *p* = 0.004], externalizing [OR: 13.333 (95% CI: 1.990–89.318); *p* = 0.008], total problems [OR: 9.500 (95% CI: 1.895–47.614); *p* = 0.006], and anxiety problems [OR: 6.333 (95% CI: 1.239–32.376); *p* = 0.027]. Other confounders analyzed including smoking during pregnancy, paternal educational level, place of residence and socioeconomic status, and had no effects on behavioral outcomes in infants aged 18 months and 2.5 years old (Tables S3 and S4).

## 4. Discussion

This study aimed to evaluate child behavioral development at 18 months and 2.5 years of age in relation to the type of infant feeding, trying to contribute to the current knowledge in this field. Our results seem to suggest that the infant formula enriched with specific functional nutrients might be related to better psycho-behavioral development in children aged 2.5 years compared to those who were fed with a standard infant formula. Interestingly, behavioral development in children who received bioactive compound-enriched infant formula during their first 18 months of life seemed to closely resemble those who were breastfed. In addition to the type of feeding in early postnatal life, behavioral development in children up to 2.5 seemed to be positively associated to higher maternal IQ and educational level.

Prenatal and early postnatal periods are dynamic and vulnerable windows for brain development. As a consequence, any adverse stimuli during these critical periods could negatively influence later mental health. Prenatal and postnatal nutritional status play a crucial role on brain growth and maturation with subsequent effects on neurodevelopment later in life [3,4,36].

Breastfeeding is the gold standard for nutrient intake during infancy, and its beneficial effects on neuropsychological development [37] and intelligence [38] have been widely reported both in children and adolescents. However, the relationship between breastfeeding and behavioral development is still a matter of research [10,39,40]. In this regard, beneficial effects of breastfeeding on behavioral development in children are reported as an additional finding from the current study: breastfed infants showed a lower risk of developing internalizing problems and better longitudinal behavior development in comparison with standard formula-fed infants, which could be explained by the fact that functional nutrients found in human milk must be strictly necessary for the optimal brain development and cognitive functions [41].

Infant formulas are currently improved by the addition of bioactive components trying to closely resemble human milk composition [42], in terms of optimal children growth and development [12,43]. For example, the effects of LC-PUFAs supplemented infant formulas on neurodevelopment, visual acuity and immune system [15,16] have been reported, but its effect on infant cognitive function seems to be modulated by confounding factors [44]. LC-PUFAs might have a potential beneficial role on brain structure [45] and function [46], due to its high concentration in prefrontal cortex. LC-PUFAs are also involved in signal transduction, neurotransmission, and neuroprotection. Interestingly, dysregulated lipid metabolism and a low dietary consumption of LC-PUFAs have been implicated in neuropsychiatric disorders, suicidal behaviors and neuroinflammation [46]. In this regard, it is important to note that the LC-PUFA amount contained in the EF (0.45% ARA and 0.32% DHA) was in the range recommended by the European Food Safety Authority, guaranteeing adequate levels of both ARA and DHA to support infants’ nutritional and developmental needs [47]. However, other bioactive compounds, including LC-PUFAs, MFGM, synbiotics (pre- and probiotics), as well as human milk oligosaccharides, sialic acid, nucleotides, or gangliosides, were included in the enriched infant formula tested in the COGNIS study. In this regard, MFGM-enriched infant formula was revealed to have positive effects on infants’ neurological development and the immune system [19,45]; likewise, the presence of synbiotics, which might contribute to the development of a healthy structural and functional gut microbiota [21,22], could be, in turn, involved in the modulation of brain development and neurocognitive function [23,24]. In fact, different probiotic supplementation studies have reported positive effects on psycho-behavior disorders at later ages. In a group of infants supplemented with *Lactobacillus rhamnosus* GG for the first six months of life, a significant reduction in the risk of developing ADHD and Asperger syndrome was observed at 13 years of age [48]. Sanaa et al. evaluated the symptoms in a group of autistic children aged five to nine before and after receiving a nutritional supplement of probiotics (*Lactobacillus acidophilus, Lactobacillus rhamnosus* and *Bifidobacteria* longum). Analysis performed showed a significant improvement in the severity of symptoms associated with autism [49]. These results point out that the effects observed in the current study are not related to a single nutrient; beneficial effects might be owed to a synergic effect of the diverse formula components.

To the best of our knowledge, the current study is the first one to show the effects of a functional nutrient-enriched infant formula on infant behavioral development. Our results show that a lower percentage of EF children developed clinical affective problems compared to SF children. Moreover, EF and BF children seemed to show similar increases in the score rate of behavioral problems. Consequently, these results need further research to understand which types of factors (hormonal, nutritional or psychological) are really influencing the risk of suffering behavioral problems.

Conversely, it is important to note that logistic regression models showed that higher maternal IQ and educational level were associated with lower behavioral and emotional problems in their offspring, regardless of the type of feeding received. Maternal educational level plays an important role in the establishment of adequate child behavioral development, which might be related to easy access to educational and social activities in those children born to higher educated level mothers, as previously reported [50]. Additionally, socioeconomic status and maternal educational level, but not breastfeeding, have been reported to influence children´s behavioral system and mental health [51]. Based on these findings, a better understanding of social, environmental, and nutritional factor effects is crucial for the development of early treatment and prevention programs of behavioral problems success. In this regard, existence of behavioral problems during early childhood, including internalizing or externalizing problems, are strongly related to the development of mental pathologies later in life, with increased rates of school dropout, substance abuse or suicide [52,53].

On the other hand, we are aware about the effect of smoking during pregnancy on behavioral problems. Behavioral problems of school-aged children born to mothers who smoked during pregnancy are well documented, promoting the early appearance of psychopathological symptoms such as attention deficit hyperactivity disorder [54], aggressive behavior [55] and autism spectrum disorders [56], among others. However, our data did not show the effect of smoking during pregnancy on behavior in children at 18 months of life.

The main strength of this study is its design as a prospective, randomized, double-blind longitudinal study, which enabled a long-term monitoring of behavioral development in preschool children. For this purpose, we used the CBCL test, a highly reliable and valid instrument for assessing behavioral problems in children. Interestingly, our nutritional intervention was prolonged in time (up to 18 months), which yielded results with added value with respect to other studies with shorter-time nutritional interventions (until six months of age) [22]. Furthermore, infant formula supplementation was based on a set of functional nutrients that could show a positive effect on child development. The COGNIS study also collected a wide range of information about sociodemographic characteristics, which were used as potential confounders, adding value to this nutritional intervention study.

However, the current study has limitations that must be acknowledged. Due to the COGNIS study design, the BF group was not randomized, thus differences in maternal IQ, educational level and socioeconomic status were found. Different studies have shown that mothers who breastfed their babies usually have higher educational level [52]. Apart from that, no data about maternal stress during pregnancy, which is a risk factor for adverse outcomes in infant development, was collected. In this line, prenatal exposure to maternal stress is associated with behavioral and mental health problems later in life [57]. Consequently, study results should be interpreted with caution and better effort should be made to understand how stress may affect offspring behavioral development. Moreover, as in other human RCT including long term follow-ups, part of the initial population enrolled in the study did not continue throughout follow-up visits. In this regard, despite the drop-out of participants at 2.5 years follow-up, the statistical power reached in the current study to detect a minimum difference of 0.8 SD in CBCL scores was 80%, enough to detect relevant differences in behavioral development between groups.

In summary, our findings show that the bioactive compound-enriched infant formula might have a beneficial effect on behavioral development in early childhood compared to those infants who received a standard infant formula. Moreover, results obtained also seem to propose that sociodemographic factors, such as maternal IQ and educational level, play a key role on child behavioral development, supporting a relationship between breastfeeding and psychosocial factors leading to better mental health and fewer behavioral problems in children [9]. Interestingly, there were no major behavioral differences between children who received EF and those who were breastfed up to 2.5 years of age. Future studies might provide an opportunity to improve infant formulas bringing them closer to the composition and functionality of human milk that may favor an optimal behavioral development of children.

## Figures and Tables

**Table 1:** Parents and children baseline characteristics depending on their type of feeding.

Parents Characteristics		18 Months				2.5 Years Old			
		SF (*n* = 47)	EF (*n* = 48)	BF (*n* =37)	*p* ^1^	SF (*n* = 29)	EF (*n* = 41)	BF (*n* = 33)	*p* ^1^
Maternal age (years)		32 (27–35)	32 (27.5–36)	34 (30–38)	0.12	33(29.5–3)	32 (29–36)	34(31–38.5)	0.27
Maternal pBMI, (kg/m^2^)		24.3 ± 3.9	24.8 ± 4.4	24.5 ± 3.2	0.86	24.2 ± 3.5	24.3 ± 4.3	24.9 ± 3.2	0.71
Maternal educational level	Primary	9(19.1%) ^a,b^	13 (27.1%) ^b^	1 (2.7%) ^a^	**< 0.001**	4 (13.8%)	6 (14.6%)	1 (3.0%)	**0.001**
	Secondary	14 (29.9%) ^a^	8 (16.7%) ^a,b^	2 (5.4%) ^b^		11 (37.9%) ^a^	10 (24.4%) ^a,b^	2 (6.1%) ^b^	
	VT	9 (19.1%)	15 (31.2%)	9 (24.3%)		4 (13.8%)	15 (36.6%)	8 (24.2%)	
	University	15 (31.9%) ^a^	12 (25.0%) ^a^	25 (67.6%) ^b^		10 (34.5%) ^a^	10 (24.4%) ^a^	22 (66.7%) ^b^	
Maternal IQ (points)		104.7 ± 14.4 ^a,b^	101.1 ± 14.2^a^	109.4 ± 13.5 ^b^	**0.030**	105.4 ± 15.5	102.4 ± 12.9	109.5 ± 14.1	0.10
Smoking during pregnancy	No	31 (73.8%) ^a^	37 (84.1%) ^a,b^	34 (94.4%) ^b^	**0.049**	21 (77.8%)	33 (82.5%)	31 (93.9%)	0.16
	Yes	11 (26.2%) ^a^	7 (15.9%) ^a,b^	2 (5.6%) ^b^		6 (22.2%)	7 (17.5%)	2 (6.1%)	
GWG (kg)		6.5 (4–10)	5 (3–9)	6.3 (4.5–9)	0.80	5 (3–8)	5 (3–8.7)	6 (4.2–8.7)	0.42
Type of delivery	Vaginal	34 (72.3%)	33 (68.8%)	27 (73.0%)	0.89	23 (79.3%)	27 (65.9%)	25 (75.8%)	0.46
	Caesarean	13 (27.7%)	15 (31.2%)	10 (27.0%)		6 (20.7%)	14 (34.1%)	8 (24.2%)	
Postpartum Depression	No	38 (80.9%)	39 (81.2%)	31 (83.8%)	0.93	22 (75.9%)	34 (82.9%)	28 (84.8%)	0.63
	Yes	9 (19.1%)	9 (18.8%)	6 (16.2%)		7 (24.1%)	7 (17.1%)	5 (15.2%)	
Paternal educational level	Primary	16 (34.8%) ^a,b^	23 (48.9%) ^b^	6 (16.2%) ^a^	**0.013**	10 (34.5%)	16 (39.0%)	6 (18.2%)	0.45
	Secondary	13 (28.3%)	6 (12.8%)	5 (13.5%)		6 (20.7%)	8 (19.5%)	5 (15.2%)	
	VT	7 (15.2%)	8 (17.0%)	11 (29.8%)		6 (20.7%)	9 (22.0%)	10 (30.3%)	
	University	10 (21.7%)	10 (21.3%)	15 (40.5%)		7 (24.1%)	8 (19.5%)	12 (36.4%)	
Paternal IQ (points)		105.9 ± 13.8	106.2 ± 15.2	107.2 ± 13.3	0.93	107.8 ± 14.1	104.8 ± 15.1	106.9 ± 12.9	0.70
Socioeconomic status	Low	N/A	N/A	N/A		6 (21.4%) ^a,b^	13 (31.7%) ^b^	1 (3.0%) ^a^	**<0.001**
	Mid–Low	N/A	N/A	N/A		14 (50.0%)	21 (51.2%)	11 (33.3%)	
	Mid–High	N/A	N/A	N/A		7(25.0%) ^a,b^	7 (17.1%) ^b^	14 (42.4%) ^a^	
	High	N/A	N/A	N/A		1 (3.6%) ^a,b^	0 (0.0%) ^b^	7 (21.3%) ^a^	
Place of residence	Urban	23 (48.9%) ^a^	14 (29.2%) ^a,b^	9 (24.3%) ^b^	**0.037**	18 (62.1%) ^a^	13 (33.3%) ^a,b^	9 (27.3%) ^b^	**0.012**
	Rural	24 (51.1%) ^a^	34 (70.8%) ^a,b^	28 (75.7%) ^b^		11 (37.9%) ^a^	26 (66.7%) ^a,b^	24 (72.7%) ^b^	
Siblings	0	21 (44.7%)	25 (52.1%)	20 (54.1%)	0.65	9 (31.0%)	16 (39.0%)	12 (36.4%)	0.79
	≥1	26 (55.3%)	23 (47.9%)	17 (45.9%)		20 (69.0%)	25 (61.0%)	21 (63.6%)	
Gestational age (weeks)		40 (39–41)	40 (39–40)	40 (39–41)	0.50	40 (39–41)	40 (37.5–40.5)	40 (38.5–41)	0.67
Newborn characteristics									
Newborn weight (g)		3350.1 ± 413.7	3241.5 ± 434.4	3395.7 ± 381.1	0.20	3344.8 ± 462.6	3297.3 ± 569.0	3374.2 ± 392.6	0.74
Newborn length (cm)		51 (49.5–52)	50.5 (49–52)	51 (50–52)	0.52	51 (49.2–52.9)	51 (49–52)	51 (50–52)	0.70
Newborn HC (cm)		34.5 (34–36)	34 (34–35)	35 (33.8–35)	0.15	35 (34–35.7)	34 (33.5–35)	35 (33.6–35.7)	0.25
Newborn Gender	Boy	27 (57.4%)	29 (60.4%)	15 (40.5%)	0.16	18 (62.1%)	25 (61.0%)	13 (39.4%)	0.06
	Girl	20 (42.6%)	19 (39.6%)	22 (59.5%)		11 (37.9%)	16 (39.0%)	20 (60.6%)	
Breastfeeding lactation (days)		10 (0–21) ^a^	11.5 (0.5–25) ^a^	420 (270–540) ^b^	**<0.001**	15 (5–43.5) ^a^	14 (2.5–29.5) ^a^	390 (270–765) ^b^	**<0.001**

Data are presented as *n* (%) for categorical data, mean ± SD for parametrically distributed data, and median (IQR) for non-parametrically distributed data. ^1^ *p*-values for overall differences between COGNIS-groups. ANOVA for normally distributed variables, Kruskal–Wallis rank-sum test for non-normal continuous variables and Chi-squared or Fisher’s exact test for categorical variables. Values which do not share the same suffix (ab) are significantly different in a Bonferroni post-hoc test. Bold: *p*-value < 0.05. BF, breastfed infants; EF, experimental infant formula; GWG, gestational weight gain; HC, head circumference; IQ, intelligence quotient; IQR, interquartile range; Mid, middle; N/A, not available (data not recorded); pBMI, pre-conceptional body mass index; SD, standard deviation; SF, standard infant formula; VT, vocational training.

**Table 2:** Association between early nutrition and CBCL clinical clusters problems in children at 18 months and 2.5 years old.

CBCL Scales			18 Months					2.5 Years Old			
		SF (*n* = 47)	EF (*n* = 48)	BF (*n* = 37)	Fisher’s Exact Test	*p* ^1^	SF (*n* = 29)	EF (*n* = 41)	BF (*n* = 33)	Fisher’s Exact Test	*p* ^1^
Internalizing Problems	Normal	41 (87.2%)	41 (85.4%)	33 (89.2%)	2.606	0.65	17 (58.7%)	33 (80.5%)	28 (84.9%)	9.516	**0.042**
	Borderline	5 (10.6%)	3 (6.3%)	3 (8.1%)			7 (24.1%) ^a^	6 (14.6%) ^a,b^	1 (3.0%) ^b^		
	Pathology	1 (2.1%)	4 (8.3%)	1 (2.7%)			5 (17.2%)	2 (4.9%)	4 (12.1%)		
Externalizing Problems	Normal	39 (83.0%)	41 (85.4%)	32 (86.5%)	4.220	0.37	22 (75.9%)	36 (87.8%)	29 (87.9%)	6.470	0.15
	Borderline	5 (10.6%)	3 (6.3%)	5 (13.5%)			1 (3.4%)	3 (7.3%)	3 (9.1%)		
	Pathology	3 (6.4%)	4 (8.3%)	0 (0.0%)			6 (20.7%)	2 (4.9%)	1 (3.0%)		
Total Problems	Normal	40 (85.1%)	39 (81.3%)	31 (83.8%)	1.659	0.83	19 (65.5%)	33 (80.5%)	28 (84.8%)	6.970	0.13
	Borderline	5 (10.6%)	4 (8.3%)	4 (10.8%)			3 (10.3%)	6 (14.6%)	2 (6.1%)		
	Pathology	2 (4.3%)	5 (10.4%)	2 (5.4%)			7 (24.2%)	2 (4.9%)	3 (9.1%)		
Affective Problems	Normal	45 (95.7%)	42 (87.5%)	35 (94.6%)	4.386	0.35	25 (86.2%)	40 (97.6%)	28 (84.8%)	8.491	**0.026**
	Borderline	2 (4.3%)	2 (4.2%)	1 (2.7%)			0 (0.0%)	1 (2.4%)	3 (9.1%)		
	Pathology	0 (0.0%)	4 (8.3%)	1 (2.7%)			4 (13.8%) ^a^	0 (0.0%) ^b^	2 (6.1%) ^a,b^		
Anxiety Problems	Normal	46 (97.9%)	46 (95.8%)	35 (94.6%)	1.948	0.94	24 (82.8%)	36 (87.8%)	29 (87.9%)	1.700	0.92
	Borderline	1 (2.1%)	1 (2.1%)	1 (2.7%)			1 (3.4%)	1 (2.4%)	0 (0.0%)		
	Pathology	0 (0.0%)	1 (2.1%)	1 (2.7%)			4 (13.8%)	4 (9.8%)	4 (12.1%)		
Perv. Develop. Problems	Normal	44 (93.6%)	43 (89.6%)	33 (89.2%)	2.027	0.78	23 (79.3%)	34 (82.9%)	32 (97.0%)	5.207	0.25
	Borderline	1 (2.1%)	3 (6.3%)	1 (2.7%)			4 (13.7%)	4 (9.8%)	1 (3.0%)		
	Pathology	2 (4.3%)	2 (4.2%)	3 (8.1%)			2 (6.9%)	3 (7.3%)	0 (0.0%)		
ADHD	Normal	44 (93.6%)	46 (95.8%)	35 (94.6%)	1.100	0.97	26 (89.7%)	39 (95.2%)	33 (100%)	3.589	0.46
	Borderline	2 (4.3%)	1 (2.1%)	1 (2.7%)			1 (3.4%)	1 (2.4%)	0 (0.0%)		
	Pathology	1 (2.1%)	1 (2.1%)	1 (2.7%)			2 (6.9%)	1 (2.4%)	0 (0.0%)		
Op. Def. Problems	Normal	45 (95.7%)	46 (95.8%)	37 (100%)	3.118	0.78	28 (96.5%)	37 (90.3%)	32 (97.0%)	2.397	0.85
	Borderline	2 (4.3%)	1 (2.1%)	0 (0.0%)			0 (0.0%)	1 (2.4%)	0 (0.0%)		
	Pathology	0 (0.0%)	1 (2.1%)	0 (0.0%)			1 (3.5%)	3 (7.3%)	1 (3.0%)		

Data are presented as *n* (%) for categorical data. Fisher’s exact test for categorical variables. ^1^ *p*-values are comparisons between COGNIS-groups. Values which do not share the same suffix (ab) are significantly different in a Bonferroni post-hoc test. Bold: *p*-value <0.05. ADHD, attention deficit/hyperactivity disorders; BF, breastfed infants; EF, experimental infant formula; Op. Def., oppositional defiant; Perv. Develop., pervasive developmental; SF, standard infant formula.

**Table 3:** Longitudinal study of CBCL scores up to 2.5 years of age in COGNIS participants.

CBCL Scales		18 Months	2.5 Years	F(df)	*p* ^1^	*p* ^2^	*p* ^3^	η_p_^2^
Internalizing Problems	SF	8.7 ± 4.6	12.6 ± 9.9 ^a^	3.041(2,98)	**0.011**	0.16	**0.047**	0.058
	EF	8.0 ± 6.7	9.0 ± 6.0 ^a,b^					
	BF	6.8 ± 5.5	7.5 ± 6.0 ^b^					
Externalizing Problems	SF	14.6 ± 6.2	16.9 ± 7.9 ^a^	3.885(2,98)	0.77	0.13	**0.024**	0.073
	EF	12.5 ± 6.9	12.2 ± 6.8 ^b^					
	BF	12.4 ± 6.6	11.1 ± 7.7 ^b^					
Total Problems	SF	37.9 ± 13.9	46.1 ± 26.3 ^a^	3.222(2,98)	0.15	0.16	**0.044**	0.062
	EF	33.0 ± 21.3	34.4 ± 17.5 ^a,b^					
	BF	32.2 ± 16.9	31.1 ± 19.6 ^b^					
Affective Problems	SF	2.6 ± 1.5	3.4 ± 2.6	0.403(2,98)	0.14	0.07	0.67	0.008
	EF	2.8 ± 2.9	2.4 ± 1.6					
	BF	2.2 ± 1.8	2.9 ± 2.2					
Anxiety Problems	SF	3.6 ± 1.7	4.7 ± 3.0	0.500(2,98)	**0.005**	0.34	0.61	0.010
	EF	3.3 ± 2.2	4.2 ± 3.0					
	BF	3.5 ± 2.2	3.7 ± 3.0					
Perv. Develop. Problems	SF	3.5 ± 2.5	4.4 ± 3.7	1.710(2,98)	0.25	0.42	0.19	0.034
	EF	3.3 ± 3.5	3.8 ± 3.0					
	BF	3.0 ± 2.6	2.8 ± 2.1					
ADHD	SF	5.8 ± 2.5	6.1 ± 2.6 ^a^	3.442(2,98)	0.11	0.06	**0.036**	0.066
	EF	4.9 ± 2.3	4.6 ± 2.7 ^a,b^					
	BF	5.2 ± 2.6	3.9 ± 2.7 ^b^					
Op. Def. Problems	SF	3.6 ± 2.1	4.5 ± 2.0 ^a^	3.630(2,98)	**0.034**	0.39	**0.030**	0.069
	EF	2.9 ± 1.8	3.5 ± 2.5 ^a,b^					
	BF	2.9 ± 1.9	2.9 ± 2.3 ^b^					

Data are presented as mean ± SD. SF (*n* = 29); EF (*n* = 39); BF (*n* = 33). *p*-values, F-values (F) and effect sizes (η_p_^2^) were obtained from a generalized linear mixed model of repeated measures: ^1^ shows differences between time points; ^2^ differences between time points according to the COGNIS groups; ^3^ longitudinal differences between the COGNIS groups. Values which do not share the same suffix (ab) are significantly different in a Bonferroni post-hoc test. Bold: *p*-value <0.05. ADHD, attention deficit/hyperactivity disorders; BF, breastfed infants; df, degrees of freedom; EF, experimental infant formula; Op. Def., oppositional defiant; Perv. Develop, pervasive developmental; SF, standard infant formula. Mean normative values: total problems = 33.5; externalizing problems = 14; internalizing problems = 9; affective problems = 2.2; anxiety problems = 3.5; pervasive developmental problems = 3; ADHD = 5.5 and oppositional defiant problems = 4 [33].

## Supplementary Materials

The following are available online at https://www.mdpi.com/2072-6643/12/12/3825/s1; Table S1: Nutritional composition of the Standard (SF) and Experimental (EF) Infant Formulas used in the COGNIS study; Table S2: Effects of SF, EF or BF on CBCL scores at 18 months and 2.5 years old in infants participating in the COGNIS study; Table S3: Association of maternal IQ and educational level, smoking during pregnancy, paternal educational level, place of residence and three COGNIS study groups with children behavioral outcomes at 18 months of life; Table S4: Association of maternal educational level, socioeconomic status, place of residence and three COGNIS study groups with children behavioral outcomes at 2.5 years old.

## Statement

The results of this article have been included in the Doctoral Thesis of A.N.-R. in the context of the Clinical Medicine and Public Health Doctoral Program at the University of Granada.
