# Supplementary material for: The Effects of an Infant Formula Enriched with Milk Fat Globule Membrane, Long-Chain Polyunsaturated Fatty Acids and Synbiotics on Child Behavior up to 2.5 Years Old: The COGNIS Study
Source: Nutrients. 2020 Dec 15;12(12):3825. doi: 10.3390/nu12123825 (PMC7765166; doi:10.3390/nu12123825)
Supplement: Supplementary file 1 [file nutrients-12-03825-s001.pdf]

Table S1. Nutritional composition of the Standard (SF) and Experimental (EF) Infant Formulas used in the COGNIS study.

	Standard Formula (SF)		Experimental Formula (EF)	
	**Initiation**	**Follow-on**	**Initiation**	**Follow-on**
	100 ml (13.5%)	100 ml (14.5%)	100 ml (13.5%)	100 ml (14.5%)
**Energy** (kcal/kJ)	69/288	70/294	68/285	69/290
**Proteins** (g)	1.35	1.8	1.35	1.8
Casein/whey (%)	40/60	50/50	40/60	50/50
**Carbohydrates** (g)	7.97	8.5	7.56	8.1
Lactose (g)	7.17	7.2	6.82	7.3
Maltodextrin (g)	0.8	1.3	0.7	0.8
**Fat** (g)	3.5	3.2	3.5	3.2
LA (mg)	579	517	569	517
α-ALA (mg)	49	45	49	45
ARA (mg)	-	-	15.8	10.2
DHA (mg)	-	-	11.2	10.2
**Gangliosides** (mg/L)	1.5	1.5	9	9
**Sialic acid** (mg/L)	82	80	105	105
**MFGM-10** (wt/wt)	-	-	10%	10%
**Nucleotides** (mg)	-	-	2.92	2.94
Cytidine-5'-Monophosphate (mg)	-	-	1.09	1.12
Uridine-5'-Monophosphate (mg)	-	-	0.88	0.9
Adenosine-5'-Monophosphate (mg)	-	-	0.41	0.41
Guanosine-5'-Monophosphate (mg)	-	-	0.27	0.26
Inosine-5'-Monophosphate (mg)	-	-	0.27	0.26
**Prebiotics**				
FOS: Inulin (1:1)	-	-	0.4	0.4
**Probiotics**				
Bifidobacterium L. infantis CECT7210 (*Bifidobacterium infantis* IM1)	-	-	1x10^7 cfu/g	1x10^7 cfu/g
Lactobacillus rhamnosus LCS-742	-	-	1x10^7 cfu/g	1x10^7 cfu/g

ARA: Arachidonic acid; cfu: colony forming unit; DHA: Docosahexaenoic acid; FOS: Fructooligosaccharides; LA: Linoleic acid; MFGM: Milk Fat Globule Membrane; α-ALA: α-Linolenic acid. Power diluted 13.5% (13.5 g infant formula made up to 100 ml with water); Power diluted 14.5% (14.5 g infant formula made up to 100 ml with water). Both standard and experimental Initiation infant formulas were prolonged from birth/2 months till 6 months of life; then, infants received corresponding Follow-on formula from 6 to 18 months of age.

Table S2. Effects of SF, EF or BF on CBCL scores at 18 months and 2.5 years old in infants participating in the COGNIS study.

CBCL Scales	18 months							2.5 years old						
	**SF (n=47)**	**EF (n=48)**	**BF (n=37)**	p^1	$P_{adj}^{\dagger}$	**F(df)**	η_p^2	**SF (n=29)**	**EF (n=41)**	**BF (n=33)**	p^1	$P_{adj}^{\ddagger}$	**F(df)**	η_p^2
Internalizing Problems	49.8±8.3	50.1±9.2	47.4±9.1	0.33	0.81	0.21(2,112)	0.00	55.1±11.3[a]	51.0±10.6[a,b]	47.8±10.8[b]	**0.035**	0.37	1.02(2,94)	0.02
Externalizing Problems	51.8±7.4	51.0±8.9	49.7±8.4	0.52	0.70	0.35(2,112)	0.01	55.2±9.2[a]	49.3±9.1[b]	47.5±10.1[b]	**0.005**	0.10	2.40(2,94)	0.05
Total Problems	51.7±7.5	51.2±10.0	50.0±9.1	0.69	0.61	0.49(2,112)	0.01	56.3±11.3[a]	51.1±9.4[a,b]	48.8±10.9[b]	**0.017**	0.27	1.37(2,94)	0.03
Affective Problems	54.8±4.6	56.5±7.9	54.5±5.3	0.35	0.74	0.31(2,112)	0.01	57.7±7.8	54.9±4.6	56.6±6.8	0.18	0.19	1.67(2,94)	0.03
Anxiety Problems	53.4±4.2	54.0±5.1	53.9±5.3	0.83	0.57	0.57(2,112)	0.01	56.9±8.6	56.1±7.4	55.4±7.1	0.75	0.98	0.02(2,94)	0.00
Perv. Develop. Problems	54.2±5.7	55.0±7.2	54.4±6.8	0.82	0.91	0.09(2,112)	0.00	57.4±8.7	56.5±6.8	54.1±4.7	0.14	0.65	0.43(2,94)	0.01
ADHD	54.7±5.6	54.0±5.5	54.9±5.2	0.72	0.44	0.83(2,112)	0.02	56.1±6.6[a]	53.7±5.1[a,b]	52.7±4.2[b]	**0.039**	0.56	0.60(2,94)	0.01
Op. Def. Problems	53.0±4.1	52.8±4.9	52.1±3.2	0.56	0.77	0.26(2,112)	0.01	55.3±5.1	53.5±5.9	52.6±4.7	0.13	0.57	0.58(2,94)	0.01

Data are presented as means±SD of typical scores. p^1 is ANOVA test for normally distributed variables. Values not sharing the same suffix (ab) were significantly different in a Bonferroni *post hoc* test. Bold: *p value <0.05.* $P_{adj}^{\dagger}$ is ANCOVA for the group differences using the univariate general linear model, including confounder factors: maternal educational level, maternal IQ, smoking during pregnancy, paternal educational level and place of residence. $P_{adj}^{\ddagger}$ is ANCOVA for the group differences using the univariate general linear model, including confounder factors: maternal educational level, socioeconomic status and place of residence. F-values (F) and effect sizes (η_p^2) were calculated by ANCOVAs. ADHD: Attention Deficit/Hyperactivity Disorders; BF: Breastfed infants; df: degrees of freedom; EF: Experimental infant formula; Op. Def.: Oppositional Defiant; Perv. Develop: Pervasive Developmental; SF: Standard infant formula.

Table S3. Association of maternal IQ and educational level, smoking during pregnancy, paternal educational level, place of residence and three COGNIS study groups with children behavioral outcomes at 18 months of life.

	**Maternal IQ**		**Maternal educational level (Secondary)**		**Smoking during pregnancy**		**Paternal educational level (Secondary)**		**Place of residence (Urban)**		**SF group**		**EF group**	
**CBCL Scales**	**OR (95% CI)**	***p*[1]**	**OR (95% CI)**	***p*[1]**	**OR (95% CI)**	***p*[1]**	**OR (95% CI)**	***p*[1]**	**OR (95% CI)**	***p*[1]**	**OR (95% CI)**	***p*[1]**	**OR (95% CI)**	***p*[1]**
Internalizing Problems	0.95(0.91-0.97)	**0.010**	0.90(0.13-6.51)	0.93	1.80(0.47-6.84)	0.39	1.30(0.11-15.22)	0.84	1.14(0.25-5.18)	0.86	0.58(0.11-3.00)	0.51	0.51(0.10-2.60)	0.42
Externalizing Problems	0.97(0.94-1.01)	0.17	1.57(0.33-7.51)	0.57	0.67(0.11-3.93)	0.66	0.53(0.15-3.57)	0.70	0.80(0.20-3.18)	0.75	1.02(0.22-4.84)	0.98	0.69(0.15-3.21)	0.64
Total Problems	0.97(0.93-1.00)	**0.048**	1.23(0.15-10.37)	0.85	0.76(0.19-3.01)	0.70	0.73(0.15-3.57)	0.70	1.14(0.36-3.59)	0.83	0.67(0.17-2.64)	0.56	0.63(0.17-2.39)	0.50
Affective Problems	0.98(0.91-1.07)	0.72	N/A	N/A	1.10(0.08-14.44)	0.94	N/A	N/A	0.33(0.04-2.83)	0.31	0.51(0.04-6.04)	0.59	1.73(0.30-10.08)	0.54
Anxiety Problems	0.98(0.92-1.05)	0.57	N/A	N/A	3.59(0.56-23.04)	0.18	N/A	N/A	1.53(0.24-9.79)	0.65	0.16(0.01-2.73	0.21	0.52(0.06-4.74)	0.56
Perv. Develop. Problems	0.97(0.93-1.02)	0.21	0.63(0.05-7.90)	0.72	0.40(0.05-3.39)	0.40	0.69(0.07-6.84)	0.75	1.24(0.24-6.52)	0.80	0.55(0.10-3.21)	0.51	0.50(0.10-2.60)	0.41
ADHD	0.95(0.90-1.01)	0.10	6.86(1.14-41.43)	**0.036**	0.74(0.08-7.18)	0.80	2.46(0.06-94.90)	0.63	0.41(0.04-3.74)	0.43	0.66(0.05-8.77)	0.75	0.69(0.06-8.51)	0.77
Op. Def. Problems	1.00(0.92-1.10)	0.93	7.75(0.61-98.24)	0.11	N/A	N/A	N/A	N/A	4.32(0.38-49.21)	0.24	N/A	N/A	N/A	N/A

[1]p-values were obtained by logistic regression analysis (Wald method). Dichotomized scales for binary logistic regression: normal and borderline/clinical pathology. Bold: *p value* <0.05. ADHD: Attention Deficit/Hyperactivity Disorders; CI: Confidence Interval; EF: Experimental infant formula; N/A: Not available; OR: Odds Ratio; Op. Def.: Oppositional Defiant; Perv. Develop: Pervasive Developmental; SF: Standard infant formula. Not available data were excluded directly by the model.

Table S4. Association of maternal educational level, socioeconomic status, place of residence and three COGNIS study groups with children behavioral outcomes at 2.5 years old.

CBCL Scales	Maternal educational level (Primary)		Socioeconomic Status (Low)*		Place of residence (Urban)		SF group		EF group	
	**OR (95% CI)**	***p*[1]**	**OR (95% CI)**	***p*[1]**	**OR (95% CI)**	***p*[1]**	**OR (95% CI)**	***p*[1]**	**OR (95% CI)**	***p*[1]**
Internalizing Problems	6.33(1.24-32.38)	**0.027**	0.40(0.02-7.34)	0.54	0.94(0.22-3.94)	0.93	2.14(0.55-8.36)	0.28	0.74(0.19-2.86)	0.66
Externalizing Problems	13.33(1.99-89.32)	**0.008**	N/A	N/A	1.81(0.46-7.20)	0.40	0.54(0.08-3.45)	0.51	0.37(0.07-1.90)	0.23
Total Problems	9.50(1.89-47.61)	**0.006**	0.61(0.04-9.99)	0.73	1.90(0.55-6.47)	0.31	1.28(0.26-6.31)	0.76	0.79(0.18-3.53)	0.76
Affective Problems	4.96(0.32-77.93)	0.25	0.92(0.03-31.82)	0.96	1.62(0.28-9.34)	0.59	0.67(0.15-3.10)	0.61	0.15(0.02-1.33)	0.09
Anxiety Problems	6.33(1.24-32.38)	**0.027**	0.54(0.02-12.77)	0.70	1.92(0.47-7.85)	0.36	0.49(0.07-3.36)	0.47	0.56(0.11-2.81)	0.48
Perv. Develop. Problems	N/A	N/A	N/A	N/A	0.49(0.13-1.87)	0.30	6.96(0.76-63.60)	0.09	7.00(0.81-60.20)	0.08
ADHD	N/A	N/A	N/A	N/A	N/A	N/A	N/A	N/A	N/A	N/A
Op. Def. Problems	N/A	N/A	N/A	N/A	0.99(0.08-12.46)	0.99	1.00(0.05-20.76)	1.00	2.15(0.19-24.71)	0.54

[1]p-values were obtained by logistic regression analysis (Wald method). Dichotomized scales for binary logistic regression: normal and borderline/clinical pathology. Bold: *p value <0.05.* ADHD: Attention Deficit/Hyperactivity Disorders; CI: Confidence Interval; EF: Experimental infant formula; N/A: Not available; OR: Odds Ratio; Op. Def.: Oppositional Defiant; Perv. Develop: Pervasive Developmental; SF: Standard infant formula. *Low socioeconomic status were defined as monthly income less than EUR 1000. Not available data were excluded directly by the model.
